# FrameD: framework for DNA-based data storage design, verification, and validation

**DOI:** 10.1093/bioinformatics/btad572

**Published:** 2023-09-15

**Authors:** Kevin D Volkel, Kevin N Lin, Paul W Hook, Winston Timp, Albert J Keung, James M Tuck

**Affiliations:** Department of Electrical and Computer Engineering, North Carolina State University, Raleigh, NC, 27606, United States; Department of Chemical and Biomolecular Engineering, North Carolina State University, Raleigh, NC, 27695, United States; Department of Biomedical Engineering, Johns Hopkins University, Baltimore, MD, 21218, United States; Department of Biomedical Engineering, Johns Hopkins University, Baltimore, MD, 21218, United States; Department of Chemical and Biomolecular Engineering, North Carolina State University, Raleigh, NC, 27695, United States; Department of Electrical and Computer Engineering, North Carolina State University, Raleigh, NC, 27606, United States

## Abstract

**Motivation:**

DNA-based data storage is a quickly growing field that hopes to harness the massive theoretical information density of DNA molecules to produce a competitive next-generation storage medium suitable for archival data. In recent years, many DNA-based storage system designs have been proposed. Given that no common infrastructure exists for simulating these storage systems, comparing many different designs along with many different error models is increasingly difficult. To address this challenge, we introduce FrameD, a simulation infrastructure for DNA storage systems that leverages the underlying modularity of DNA storage system designs to provide a framework to express different designs while being able to reuse common components.

**Results:**

We demonstrate the utility of FrameD and the need for a common simulation platform using a case study. Our case study compares designs that utilize strand copies differently, some that align strand copies using multiple sequence alignment algorithms and others that do not. We found that the choice to include multiple sequence alignment in the pipeline is dependent on the error rate and the type of errors being injected and is not always beneficial. In addition to supporting a wide range of designs, FrameD provides the user with transparent parallelism to deal with a large number of reads from sequencing and the need for many fault injection iterations. We believe that FrameD fills a void in the tools publicly available to the DNA storage community by providing a modular and extensible framework with support for massive parallelism. As a result, it will help accelerate the design process of future DNA-based storage systems.

**Availability and implementation:**

The source code for FrameD along with the data generated during the demonstration of FrameD is available in a public Github repository at https://github.com/dna-storage/framed, (https://dx.doi.org/10.5281/zenodo.7757762).

## 1 Introduction

The world is generating data faster and in larger quantities than ever before, raising concerns that traditional storage technologies will not scale to keep up with demand. In the search for new technologies, DNA has gained broad interest due to its superior density and longevity compared to magnetic tape and hard disk drives. Since the early work of Church *et al.* (2012) and Goldman *et al.* (2013) demonstrating the ability to store information in DNA strands using modern DNA technology, there have been a range of studies answering important questions such as data addressability (Bornholt *et al.* 2016, Organick *et al.* 2018, 2020, Tomek *et al.* 2019, 2021, Lin *et al.* 2020), synthesis efficiency (Anavy *et al.* 2019, Choi *et al.* 2019, Antkowiak *et al.* 2020), DNA reusability (Tomek *et al.* 2019, Lin *et al.* 2020), error rates associated with a variety of techniques (Grass *et al.* 2015, Organick *et al.* 2018, Tomek *et al.* 2019, Matange *et al.* 2021), and the density that can be achieved in DNA molecules (Goldman *et al.* 2013, Erlich and Zielinski 2017, Anavy *et al.* 2019, Choi *et al.* 2019, Antkowiak *et al.* 2020). This vast knowledge base of DNA-based data storage comes with an equally expansive space of possible implementation approaches for which little if any consensus has been reached. Compounding the problem of choosing any one approach is the fact that there is a lack of common infrastructure that enables the comparison of these designs in a fair and reproducible manner.

To address the growing need for tools to analyze and compare DNA storage systems, we present FrameD, a software framework for designing, verifying, and validating DNA storage system designs. FrameD is not a library of every conceivable error correction algorithm, instead, it provides a fault-injection-based test bed in which DNA storage systems can be evaluated. Constructing FrameD requires several considerations. One being FrameD’s flexibility in terms of what DNA storage systems can be represented. To address this issue, we use current literature to inform the construction of a model that can be used as a basis in which a range of DNA storage systems can be expressed. With this model, we are able to implement an execution back-end that executes a set of encoding steps that adhere to the model’s interfaces. Thus, for an encoding to be used in FrameD, a user need only follow the interface specification. This execution model back-end also provides transparent support for necessary bookkeeping steps like DNA strand indexing and dropout inference, allowing the user to focus on the details of their algorithms.

Another issue that needs to be considered when simulating DNA storage systems is computational scale issues that arise from several sources. One source is the size of the possible parameter space of interest with regards to an encoding/decoding algorithm, as exploring combinations of parameters can easily lead to exponential growth in the number of experiments. Another source of computational scale arises from the necessity to perform fault injection experiments 1000’s of times to achieve narrow confidence intervals on key outcomes such as strand and file decode rates. Compounding each source of computational overhead is the scale of sequencing data that needs to be processed. To support scalability, FrameD utilizes batch jobs to parallelize individual configuration simulations and Message Passing Interface (MPI) to parallelize units of work within those batch jobs like strand decoding and fault injection iterations. FrameD implements the parallelization support transparently such that users do not need to manage parallelization communication. Instead, the user just specifies their configurations and the computational resources to allocate to each.

We demonstrate the utility of FrameD by performing a comparison between three designs across two error models representing different sequencing technology. We evaluate 240 total configurations, generating a total of 654 million fault-injected DNA strands, and analyze the read and write density trade-off between the three designs. Our results show that the optimal design approach depends on the designer’s read and write cost targets and the target sequencing technology, and bolsters our claim that a common simulation infrastructure is needed.

## 2 The case for DNA storage simulation infrastructure

Before discussing details of FrameD, we present a study of current literature to further motivate the need for a DNA simulation infrastructure and to understand the basic components that such an infrastructure will need to support. For our review, we choose 13 previous works that implement end-to-end DNA storage systems. We selected these works because they are representative of different approaches that have been taken since the revival of DNA data storage started by Goldman *et al.*’s work. Thus, we should be able to make conclusions about consistent approaches taken in DNA storage design, while also being able to account for the inclusion of novel techniques from each individual work. Detailed organization of these works is presented in Supplementary Table S1.

We find that transformations applied to information can be organized hierarchically in two levels. At the first level, we identify two broad categories we refer to as “**Single Strand”** and “**Multi-Strand”** transformations. Single Strand transformations focus on processing information stored in a single molecule of DNA, while the Multi-Strand processes relate to processing information stored within a group of molecules.

Under the Single Strand category, we found three typical transformation steps: “**Binary Transformation”**, “**Transcoding”**, and “**Functional Site Encoding”**. Each of these sub-categories modify the data present on a single DNA molecule in their own way. A Binary Transformation modifies the raw digital information before it is represented as DNA molecule. Such modifications typically included parity checks (Bornholt *et al.* 2016), Reed–Solomon codes (Grass *et al.* 2015, Antkowiak *et al.* 2020), and base conversions from the typical Base-2 binary representation of digital information to a numerical base that may be more convenient for a certain Reed-Solomon field (Grass *et al.* 2015, Anavy *et al.* 2019). The Transcoding category consists of processes that represent the digital source information in terms of a DNA molecule. While transcoding can be as simple as a base-conversion to base-4 (Antkowiak *et al.* 2020), approaches typically consider constraints such as GC balance (Yazdi *et al.* 2017, Press *et al.* 2020) and homopolymers (Goldman *et al.* 2013, Bornholt *et al.* 2016, Organick *et al.* 2018, Tomek *et al.* 2019), yielding a range of options with different error correction and density properties. The final Single Strand pass, Functional Site Encoding, is not inherently dependent on the raw information stored but instead includes DNA substrings in the stored molecules to facilitate functionality. Functionality encoding includes adding primers for polymerase chain reaction (PCR) random access (Bornholt *et al.* 2016, Organick *et al.* 2018, Tomek *et al.* 2019), T7 promotor sites for RNA transcription (Lin *et al.* 2020), and restriction sites for DNA fragmentation (Tomek *et al.* 2021).

Under the Multi-Strand category, we determined three distinct processing steps: “**Outer Code”**, “**Consolidation”**, and “**Reconstruction”**. The Outer Code step is similar to the Binary Transformation step of the single strand category, except error correction codes like Reed–Solomon are applied using the data of a group of strands so that errors can be corrected using information dispersed across DNA molecules (Organick *et al.* 2018, Press *et al.* 2020, Tomek *et al.* 2021). This error correction technique is crucial for dealing with the occurrence of missing DNA molecules, a common error mode of DNA storage systems (Bornholt *et al.* 2016, Organick *et al.* 2018, Press *et al.* 2020). Another issue that a DNA storage system design must address is the reconstruction of the order of information. Representing arbitrarily large sets of information requires storing subsets of information on individual DNA molecules because synthetic DNA of arbitrary length is not feasible to synthesize. Provided mixtures of DNA molecules are not guaranteed to be sequenced in any particular order, a Reconstruction strategy is needed to map a DNA molecule to its place in the complete dataset. Because of its optimality regarding density (Heckel *et al.* 2017), an “**indexing”** strategy that stores an ordering integer in each strand is a common approach (see Supplementary Table S1). Lastly, because DNA storage systems typically generate multiple copies of each transcoded DNA molecule by way of synthesis, sequencing, or amplification (Bornholt *et al.* 2016, Yazdi *et al.* 2017, Organick *et al.* 2018, Tomek *et al.* 2019), a processing step which we call Consolidation is required to generate one final representative of the information of a stored DNA molecule. This can be as simple as detecting and removing bad strands using error correction until finding a valid strand (Goldman *et al.* 2013, Bornholt *et al.* 2016, Tomek *et al.* 2019, Press *et al.* 2020), or bioinformatics tools such as multiple sequence alignment (MSA) algorithms can be employed to determine a consensus sequence (Yazdi *et al.* 2017, Organick *et al.* 2018, Antkowiak *et al.* 2020).

From this discussion, we can immediately see that the transformation of information within a DNA storage system can be described by a small set of general categories. This indicates that an infrastructure which provides support for routing this information between these categories can be effective in representing many unique DNA storage systems.

Given this observation, we consider how prior works in DNA storage simulation support general encoding and simulation environments. DNA-Storalator is a DNA storage system simulator that focuses on evaluating clustering and reconstruction algorithms that work to construct a representative DNA strand from a cluster of noisy versions. However, DNA-Storalator does not offer support for the evaluation of Outer, Binary Transformation, or Transcoding codecs (Chaykin *et al.* 2022). Furthermore, the only error models supported are those which are generated by wet-lab experiments (Sabary *et al.* 2021). While these may be accurate for a given DNA storage system implementation, allowing for user-defined error models allows for testing codecs over a wider set of cases. DNAssim offers flexible fault and coverage models, along with outer code evaluation. However, DNAssim does not consider the complex space of inner code design and chooses to only use binary to base-4 conversion as its transcoding method (Marelli *et al.* 2023). Another simulator is DeSP; however, it is only a tool that provides error injections and strand distribution changes, and so it is not a framework for evaluating different encoding designs (Yuan *et al.* 2022). In the following sections, we discuss how FrameD provides a framework in which a rich space of encodings can be evaluated using flexible error models in a scalable environment.

## 3 FrameD

**Figure 1.**
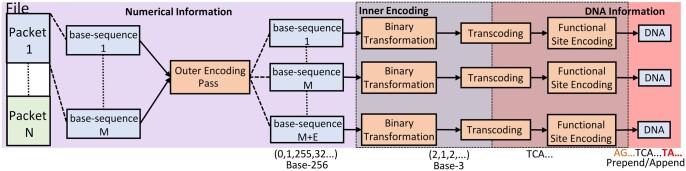
Model used by FrameD to represent encoding for DNA storage systems. Included in this figure is an example of the state of information throughout the pipeline, where initially data is in its original byte representation, then converted to base-3 and subsequently DNA, and finally stored with prepend/appended DNA strings typically done for PCR primer functionality.

We leverage categorical overlap of designs by understanding that there is a logical ordering in which the information transformations can be applied. This ordering is illustrated in Fig. 1, where information flows from left to right. First, information in a file is broken into contiguous pieces called “**packets”**. Packets serve as the scope for the outer encoding, allowing for designers to choose a granularity for the outer error correction algorithm. Before the outer code is applied, the packet is broken down into “**base-sequences”**, contiguous sections of information that will be stored on each DNA molecule. A packet’s base-sequences are then processed by the outer code, which generates indices automatically for each base-sequence while also adding additional error correction base-sequences. FrameD defaults to basic incremental integers for indexing, but provides the designer the interface to implement special indexes like the Luby Seed for Fountain codes. Each indexed base-sequence is passed through the “**inner-encoder”**, a series of steps consisting of the Single Strand operations.

The decode phase is mostly identical to encoding, except that the transformations made by each pass are reversed. However, decoding must have a mechanism to deal with multiple copies per encoded strand. We found two approaches that can be taken. One is to first cluster the DNA strands input to the decoder based on similarity scores like edit distance or using a MinHash-based approaches (Rashtchian *et al.* 2017, Organick *et al.* 2018, Antkowiak *et al.* 2020). Then MSA algorithms, such as Muscle, can be used to aggregate information across strands and help resolve errors through consensus voting (Edgar 2004, Yazdi *et al.* 2017, Antkowiak *et al.* 2020). To account for these approaches, we provide users the ability to add MSA and clustering steps to the pipeline before the inner encoding is reversed. This process is outlined in the Supplementary data. Another approach is to consolidate the strands after completing the inner code, throwing out strands that may violate error checks, and coming to a consensus on the digital representation of information. FrameD supports the use of either approach, or even both.

The pipelined approach of FrameD provides DNA storage system designers several benefits. By implementing the routing of information between components, the designer can focus on their algorithms as long as it adheres to the pipeline’s information transformation interface. Further, by breaking larger steps, e.g. inner code, down into smaller sub-components, a new unique inner code can be constructed by modifying just a single component without needing to re-implement the other algorithms that constitute the inner code. For example, one could change their Binary Transformation pass while keeping the same implementations of the Transcoding and Functional Site Encoding passes. As long as the new component adheres to its prescribed interface given the type of transformation it performs, no other work is required outside of implementing the new algorithm.

Figure 1 shows only three components within the inner encoding and one outer code component; however, we point out that both can consist of an arbitrary number. That is, Outer Codes, Binary Transformations, and Functional Site Encoding can be cascaded an arbitrary amount. For example, one may have two binary transformations, one to modify the numerical base representation of data and another one to convert to a Reed–Solomon code. We leave out Transcoding from this list, since transcoding binary information to DNA can only occur once. Details on how indexing is supported in cascaded Outer Codes is discussed in the Supplementary data. FrameD also leverages transformation type interfaces to provide validation checks for the designer to ensure that there is a logical arrangement of components.

### 3.1 Fault injection workflow

FrameD includes a simulation tool based on a fault injection methodology for evaluating DNA storage system designs. Fault injection simulation provides several benefits over the analytical model. Fault injection experiments provide the ability to quickly estimate properties of specific steps of the encoding/decoding processes. For example, fault injection experiments can determine a rate at which strands can be successfully decoded using a specified error model. This information can be used to verify analytical results or to derive parameters for a full-scale DNA storage system. Fault injection models are also more flexible, where changing an encoding or error model can make deriving a new analytical model difficult. Lastly, a fault injection framework exercises actual algorithm implementations against strands with errors in them, allowing for benchmarking and debugging that an analytical model cannot provide.

**Figure 2.**
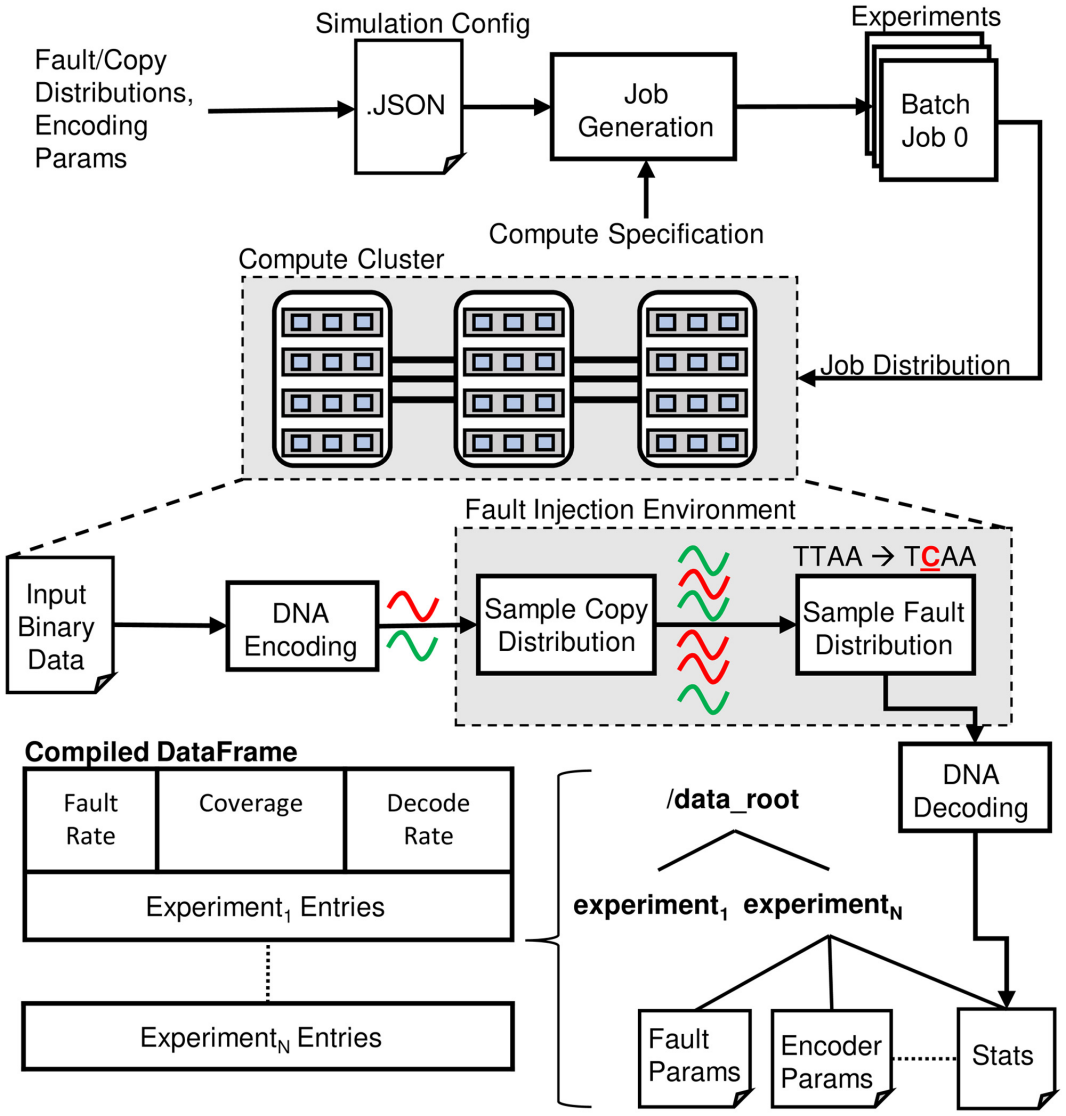
End-to-end workflow of performing fault injection simulation using FrameD.

Figure 2 overviews the workflow of using FrameD for fault injection. First, a user develops a JSON configuration file which includes a path to the binary data to be converted to DNA, along with details of three general categories of parameters that control simulation behavior: (i) encoder/decoder parameters that configure the behavior of encoding/decoding components, (ii) fault distribution parameters that configure the simulated channel’s error model, and (iii) copy distribution parameters that configure the model used to represent strand copies that arise in the storage system. Within this configuration file, users can specify a parameter sweep to explore combinations of parameters such as error rate and inner code rates, and the fault injection tool will generate individual simulation jobs (batch jobs) for each unique parameter combination.

During the evaluation of a simulation job, the input binary data is passed through the encoder to generate a library of synthetic DNA strands that subsequently sample the copy and error model distributions. The noisy set of strands passes through decoding, during which an attempt to reconstruct the original file is made, and information on errors and their locations are captured.

The output of fault injection consists of a set of files placed in a unique directory for each unique parameter combination. This set of files specifies the parameters that were used for the experiment. This allows us to keep records of all parameters used for all experiments easily. In addition to these files, a statistics file is output by the simulation, aggregating counters that are used to track events during simulation such as decode failures, location of byte-errors within strands, location of base errors within strands, etc. FrameD allows statistics to be largely user-defined so that appropriate statistics can be chosen for a given experiment. (We do not list every statistic collected, since they are easy to change.) In the following section, we outline how this is done in FrameD. Our documentation provides a tutorial on fault injection using FrameD.

### 3.2 Generating statistics

The fault injection tool of FrameD captures general system-level information out of the box such as file decode rate and total byte-errors in the final file. Designers can also further leverage FrameD to generate custom statistics of a strand’s information at various points of the pipeline. The mechanism to achieve this is called a **probe**. A probe is a special pipeline component that does not modify information, but can interrogate the state of strands as they are encoded/decoded. To calculate error rates, a probe can capture the state of the information as it is encoded. This snapshot of the information can then be used as a comparison point during decoding to calculate statistics. Such statistics can be versatile and cover a wide range of error types including errors within the bases of a strand and the errors within bytes after decoding strands. A detailed example is provided in the Supplementary data.

### 3.3 Analyzing NGS data

While this paper focuses on the *in silico* fault simulation tool of FrameD, we recognize that users of this framework would likely want to leverage the probes they created for fault injection simulation to analyze sequencing data from real experiments. In addition to the fault injection tool, we provide a tool that evaluates FrameD pipelines against next-generation sequencing (NGS) data. This additional tool allows developers the opportunity to determine which encoded DNA strand that a sequencing read originated from. This is something that is not known *a priori* and has to be computed since determining errors and their positions requires baseline information to compare against. Computing this mapping can be as simple as pairing a decoded index with the sequencing read identifier, or by computing the best alignments to a known set of strands (Sabary *et al.* 2021). Our codebase includes a mapping probe to perform this analysis along with detailed documentation on how to leverage it within FrameD for NGS data analysis.

### 3.4 Handling computational scale

**Figure 3.**
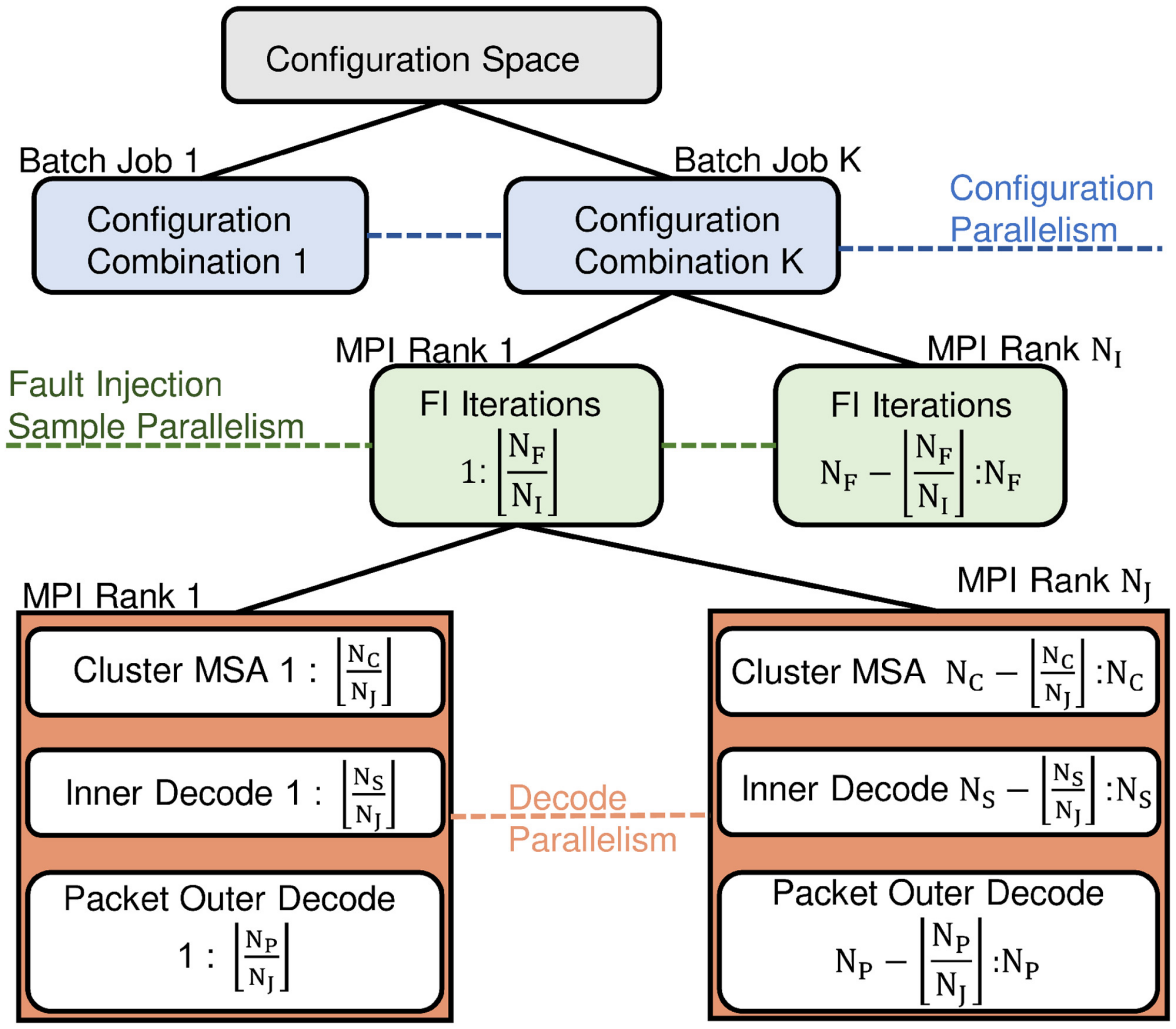
Hierarchical parallelism leveraged by FrameD. Items grouped in the same level, e.g. MSA and inner decode, represent work that can be done in parallel using the same MPI ranks. $N_{F}$, $N_{C}$, $N_{S}$, and $N_{P}$ represent the total number of fault injection iterations, clusters, strands, and packets, respectively.

When performing fault injection simulations, and decoding strands from real sequencing data, computational scalability quickly becomes an issue. FrameD enables scalability by identifying parallel units of work within the general flow of information in the framework. This allows any design to leverage paralellization transparently since FrameD can handle all communication of data. FrameD identifies parallelizaiton at three levels shown in Fig. 3.

In the first level, FrameD creates and submits individual batch jobs that can be processed by high-performance computing (HPC) workload managers like the **“Slurm Workload Manager”** or **“IBM Spectrum LSF”**. Within each batch job, FrameD can be configured to allocate compute resources in the form of MPI ranks to both fault injection iterations (second level) and work done during fault injection simulations like decoding individual strands, packet outer codes, and sequence alignment (third level). Allocation is up to the user. A user may allocate more MPI ranks to fault injection iterations if individual decode tasks are small, or the user may allocate most ranks to decoding to deal with sequencing data or to benchmark their pipeline. We utilize MPI at these levels due to its ease of communication and scalability. By targeting these clear large-grain units of work we can reach large numbers of computational cores while also keeping those resources busy.

We recognize that there may be other user-defined opportunities for parallelization. For example, consolidating DNA strands using a clustering approach may benefit from MPI-based parallelization (Rashtchian *et al.* 2017). Because communication patterns are specific to such algorithms, we provide the user with the MPI communicator that is allocated to the given fault injection iteration. This allows the user to implement their own custom parallelization if so desired. See Supplementary Figure S4 for FrameD’s communication patterns.

While FrameD aids in scaling the number of strands and fault injection experiments performed, we point out that the rate in which information will be decoded/encoded will be greatly influenced by the chosen algorithms and their implementation details. A major implementation detail impacting performance is the chosen language, and while FrameD is fully implemented in Python, the implementation of a component can be in any language as long as it has an interface to Python. We find this to be an acceptable strategy since the infrastructure of FrameD is only focused on moving information between components that perform a bulk of the computation, which can be handled by a higher performing language.

## 4 Choosing designs with FrameD

To demonstrate FrameD, we perform an analysis that serves as an example of how a DNA storage system designer may use the framework to determine the most cost-effective approach from a set of choices. In our example, we consider three different pipelines and two different error channel models, which are detailed in Table 1. Our encoder configurations are based on approaches taken in existing literature, and each leverages sequencing depth and resolves errors within a strand differently. The **RS** pipeline is representative of approaches that utilize conventional Reed–Solomon inner-error correction that deal with errors within strands post clustering and MSA (Antkowiak *et al.* 2020). Pipeline **“HEDGES”** contrasts with “**RS”** by directly resolving base errors within a strand with a convolutional code (HEDGES) without applying MSA first (Press *et al.* 2020). Lastly, we consider an approach, “**HEDGES-MSA”**, that combines information aggregation of MSA with the convolutional inner encoding to remove remaining errors. For all MSA operations, we utilize Muscle (Edgar 2004). Provided each approach leverages error correction and sequencing depth differently, we are interested in studying whether or not there are benefits as the sequencing depth and error rate of the system evolve.

**Table 1.** Table of simulated parameters.^a^

Pipeline Name	Index length	Binary transformation	Transcoding	Outer code	MSA	Depth	Inner configuration	Error model
**“RS”**	4 bytes	Randomize, $RS(2^{8})$	$\Psi (B_{2}^{2};D_{4}^{1})$	$RS(2^{8})$	Muscle	[3–30]	(1,55), (9,47) (14,42), (28,28)	i.i.d.(1,5,10%), DNArSim
**“HEDGES”**	4 bytes	N/A	HEDGES	$RS(2^{8})$	N/A	N/A	(0.167,4), (0.25,9) (0.5,24), (0.75,39)	i.i.d.(1,5,10%), DNArSim
**“HEDGES-MSA”**	4 bytes	N/A	HEDGES	$RS(2^{8})$	Muscle	[3–30]	(0.167,4), (0.25,9) (0.5,24), (0.75,39)	i.i.d.(1,5,10%), DNArSim

a For “**RS**” pipelines, the inner configuration tuples (x,y) indicate a $RS(2^{8})$ configuration of *x* redundant bytes per strand and *y* data bytes per strand. Similarly, for the “**HEDGES**” configurations, an (x,y) tuple indicates a code rate of *x* and bytes per strand of *y*.

$RS(2^{8})$ indicates a Reed-Solomon code over the field $2^{8}$, for the **“RS”** pipeline Randomize means we randomize binary information prior to the Reed-Solomon inner-error correction, and $\Psi (B_{2}^{2};D_{4}^{1})$ denotes a map between binary integers of length 2 ($B_{2}^{2}$) and DNA strings of length 1 ($D_{4}^{1}$).

DNA storage system designers are also faced with various technologies for writing/reading DNA, each with different error characteristics which can impact decoder choice. To demonstrate the evaluation of multiple error channels for the same pipelines, we consider two commonly used error models. One is a simple independent and identically distributed (i.i.d.) model for insertions, substitutions, and deletions. This model is typically used when considering NGS DNA readout (Press *et al.* 2020, Yuan *et al.* 2022). However, it has been shown that an i.i.d. channel does not well represent nanopore errors due to the lack of support for burst errors (Hamoum *et al.* 2021). To study whether burst errors may change the choice of decoder, we also consider a publicly available model, DNArSim (https://github.com/BHam-1/DNArSim), which describes conditional error probabilities that are derived from real sequencing data.

A key piece of FrameD that enables our analysis is parallelism support which allows us to simulate 240 unique pipeline configurations, totaling over 650M fault-injected DNA strands, in a reasonable time frame on a HPC cluster. All simulations were completed within 4 days on an HPC cluster with specs outlined in Table 2. To verify that our infrastructure aids in scaling to larger simulations, we perform scalability experiments on one pipeline configuration with the core type fixed to **“Intel Xeon Gold6226R”**. We picked the “**RS (9,47)”** pipeline with read depth 25× and fault rate 10%. For this study, we simulate the pipeline only 368 times, and for a single core, we measured 20 325 s to complete the simulation. Because 1 MPI rank is allocated for scheduling, 31 additional compute ranks will populate all cores of a node. In this case the experiment finishes in 802 s (25.3× speedup). In the previous cases, we are able to exclusively use a node with no outside job interference; however, for multiple node runs we are not due to our cluster being a shared system. For 92 compute ranks, we measured a 65.99× speedup. These speedups indicate that FrameD is scalable.

**Table 2.** Overview of HPC system used for simulations, parallelization parameters, and simulation characteristics.^a^

Node architecture	2 Intel Gold 6226/6130 192 GB RAM per node
Number of batch jobs	240
MPI ranks to parallelize fault injection iterations	128
Fault injection iterations/pipeline	1024
Total strands generated	654.7 million
Binary data/fault injection run	2.78 kB
Minimum inner code samples	51 200

a MPI parallelization was utilized for only fault injection iterations.

We note that FrameD only provides the infrastructure for scalability, and that efficiently executing parallel units of work requires understanding the computational resources needed by each rank. For example, developers should be aware if utilizing all cores of a node exhausts all memory when parallelizing multiple instances of the inner code, and thus allocate ranks to their compute system appropriately. In our studied pipelines, we did not observe this need, however.

This experiment also relies on other properties of FrameD. Importantly, the error model modularity allows us to apply multiple error models to each pipeline, and allows us to adopt significant portions of DNArSim with minor modifications to fit FrameD’s interface. This demonstrates FrameD’s ability to incorporate existing model implementations. Probes also play a major role in this analysis by providing decoding success rates of inner-error correction algorithms, a key value when determining storage system cost.

In all experiments, we control for strand length by modulating the number of bits of each base-sequence when we change the density of the encoding. This ensures that all designs fall in a strand length space that is reasonable given the practical limitations of DNA synthesis technologies (Bishop *et al.* 2017). In our experiments, strand lengths fall in the range of 240–242 bp. We use ideal clustering in our experiments to isolate MSA error correction from approximations made by clustering algorithms (Rashtchian *et al.* 2017, Antkowiak *et al.* 2020).

### 4.1 Comparing pipelines

We compare pipelines by calculating the read and write code rates required for each pipeline to meet a target Mean Time to Failure (MTTF) of 10^9^ accesses on 1 MB of data. We refer to these rates in terms of read and write density, each with units bits/base, and calculate them as the ratio of total bits read/written to the total number of bases read/written. Given that synthesis and sequencing cost is proportional to the number of bases, these metrics allow for technology-independent cost comparisons. A higher density is better. Write density is derived from the redundancy allocated to the inner and outer code. While read density considers encoding redundancy, it also considers the number of copies for each strand that were sequenced. We point out that the densities used for comparison are different from those provided in Table 1 for the inner codes. While the rate of the inner code influences the success rate of decoding individual strands for a given channel error, additional outer code error correction is required to develop a robust system to meet reliability targets. Thus, our final code rate is a single metric that factors in both the code rate of the inner codes shown in Table 1, and the code rate necessary for the outer code after measuring strand decode probabilities from fault injection. Detailed calculations can be found in the Supplementary data section.

With this analysis relying on estimating the decode rate of strands through the inner code, there must be enough inner code samples to build reasonable confidence for these rates. Controlling for a consistent strand length across inner encoding densities leads to some pipelines simulating more strands given a constant input binary file. However, we ensure that each inner code is simulated against at least 51k fault-injected strands.

Lastly, our analysis does not consider the impact on the outer code from physical strand dropouts that can arise throughout the DNA storage life cycle (Bornholt *et al.* 2016, Organick *et al.* 2018, Tomek *et al.* 2019). FrameD supports dropout modeling, but our focus in this case study is on the inner code’s ability to cope with errors in the channel. Thus, the outer code is entirely provisioned based on how well each inner code is able to decode strands without error. However, our design choices hold when factoring in dropouts if assuming a fixed dropout rate per pipeline.

## 5 Results

**Figure 4.**
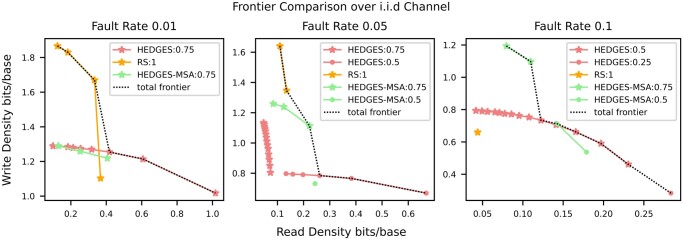
Optimal density design points for the three studied pipelines across the three studied i.i.d. error rates, 1%, 5%, and 10%. The dotted black line represents the total frontier composed of points from all of the studied pipelines. Each pipeline configuration is labeled by its inner code redundancy.

To visualize the comparison of pipeline designs across sequencing depths, we plot the Pareto front for each pipeline with respect to read and write density. A Pareto front provides the design points that optimally trade off one cost for another such that a point is included if it provides an improvement in at least one cost. If a configuration offers no benefit, it is left out. Thus, every point in Table 1 will not make it to the frontier. The pipeline frontiers for the i.i.d. error model for three error rates are shown in Fig. 4. In this figure, each pipeline has a different color, and a point shape represents a configuration of the inner code of the pipeline. The black line connecting points represents the complete frontier across all pipelines.

For the lowest fault rate (1%), we find that the “**RS”** pipeline with 1 redundancy byte for error detection provides the best write density. This configuration indicates that MSA is able to resolve a majority of errors. However, at a read density of 0.4 this pipeline’s write density drops and gets overtaken in optimality by the “**HEDGES”** pipeline. This happens because MSA alone is not able to keep up with the HEDGES code’s error correction at lower read depths, requiring the “**RS”** pipeline to use considerably more outer encoding overhead. Interestingly, adding MSA to an inner code is not always best as shown by “**HEDGES”** enveloping “**HEDGES-MSA”**. The reason stems from the HEDGES code’s high decode rate of single strands at this error rate such that it is more likely to decode a strand by applying the code multiple times rather than aggregating the information in MSA.

As the i.i.d. rate increases, MSA-based approaches become more prominent. For example, when the error rate is 5%, “**HEDGES-MSA”** outperforms “**HEDGES”** for the same inner code configuration. This is because it is now more cost-effective to use sequencing depth to reduce the per-base error rate with MSA, rather than applying the HEDGES code individually to each sequenced copy. The same occurs for a 10% error rate. Still, a pattern emerges where a non-optimal “**HEDGES-MSA”** approach becomes enveloped again by the “**HEDGES”** pipeline with the same configuration. We conclude from this that the optimality of using HEDGES with or without MSA is highly dependent on the error rate of the storage system, a conclusion a designer will not be able to come to without a simulation framework like FrameD.

A pattern that emerges for “**HEDGES”** configurations is that when read density is increased by decreasing sequencing depth, at a certain point it no longer becomes cost-effective due to ballooning outer code overhead, making lower density inner codes preferred. This can be seen for a fault rate of 5% between “**HEDGES:0.75”** and “**HEDGES:0.5”**. However, this is not the case for “**RS”**, as no configurations that utilize less dense Reed–Solomon codes for error correction appear in Fig. 4. This indicates that Reed–Solomon as an inner code is ineffective against insertions and deletions. We demonstrate this further in the Supplementary data.

**Figure 5.**
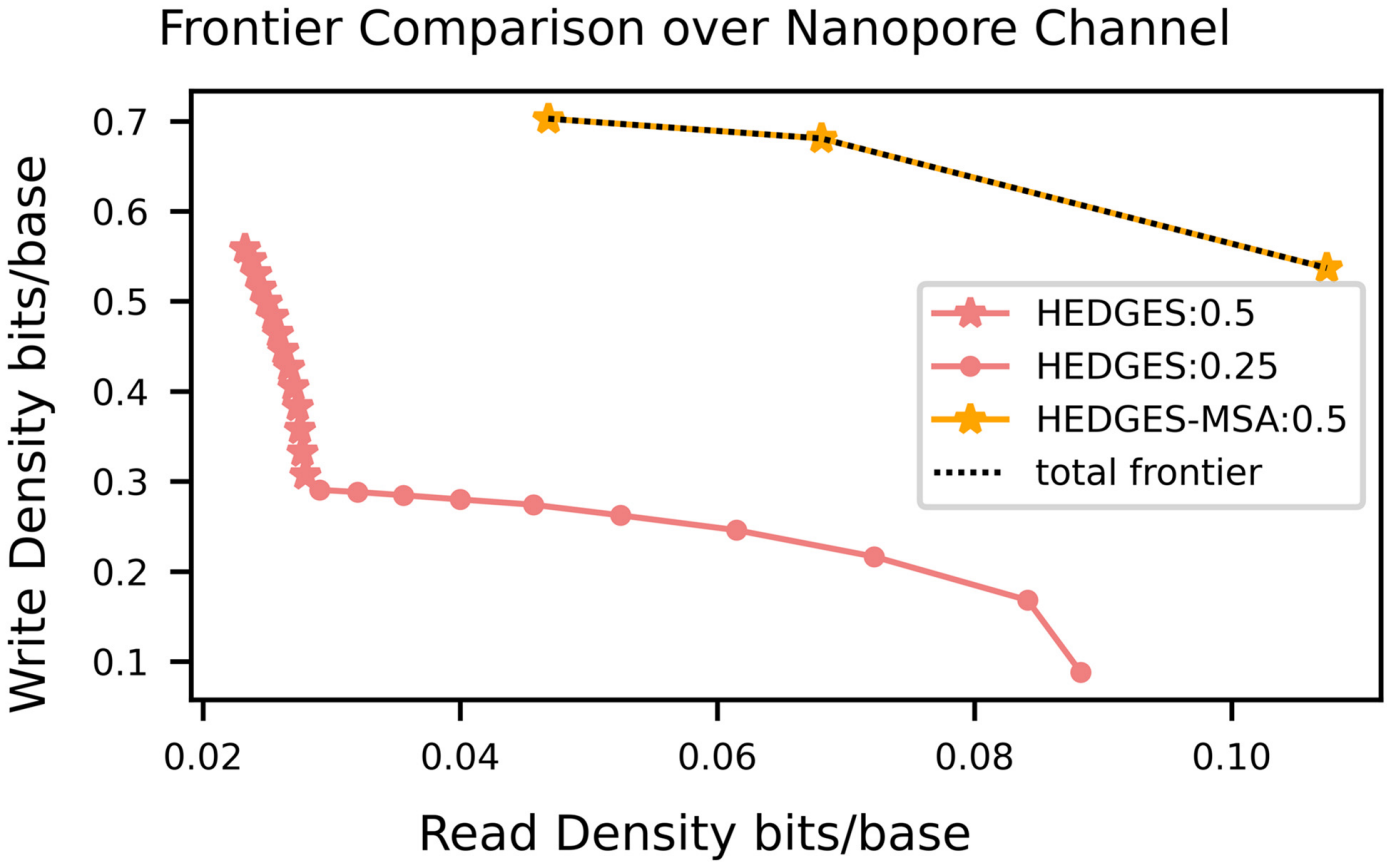
Optimal frontiers obtained when the error injection model is based on nanopore sequencing technology.

Figure 5 compares the three pipelines for nanopore-based fault injection. In contrast with the i.i.d. frontier, the complete frontier consists of points only from “**HEDGES-MSA”**. The main driving force of this is that nanopore sequencing has a higher frequency of burst errors compared to the i.i.d. model. Burst errors generate a decoder mismatch with the HEDGES code since this algorithm relies on guessing errors based on an i.i.d. error model. Thus, the HEDGES code experiences a large decode rate decrease unless MSA is applied beforehand to help resolve bursts. Another interesting component of Fig. 5 is that there is no configuration that just relies on MSA to resolve errors. These results showcase that with FrameD designers are able to define weaknesses in decoding algorithms and determine better pipeline combinations when faced with designing for different sequencing devices.

## 6 Conclusion

We have shown that as work continues in the area of DNA storage systems there is an increasing number of unique pipelines that overlap in the components that they use. We introduce FrameD to address the void of tools available to the DNA data storage community, enabling the modularization of common algorithms and integrating fault injection models to provide a basis for fair system comparisons. Because of its foundation in the literature of DNA storage systems, FrameD provides designers with the ability to simulate a wide variety of storage systems. FrameD also provides transparent support for the parallelization of computational units of work such as individual strands and fault injection iterations, enabling the use of scalable high-performance computing systems. These features are demonstrated in our analysis of three pipelines that utilize the same components in different combinations across two error models representing different sequencing devices. In our analysis, the optimal pipeline choice and configuration depends both on the cost targets set by the designer and the target sequencing device. This highlights the basic need for DNA storage designers to have tools that can compare designs across a range of environments.

## Supplementary Material

btad572_Supplementary_Data

## References

[btad572-B1] Anavy L , Vaknin I, Atar O et al Data storage in DNA with fewer synthesis cycles using composite DNA letters. Nat Biotechnol 2019;37:1229–36. 31501560 10.1038/s41587-019-0240-x

[btad572-B2] Antkowiak PL , Lietard J, Darestani MZ et al Low cost DNA data storage using photolithographic synthesis and advanced information reconstruction and error correction. Nat Commun 2020;11:5345. 33093494 10.1038/s41467-020-19148-3 PMC7582880

[btad572-B3] Bishop B , Mccorkle N, Zhirnov V. *Technology Working Group Meeting on Future DNA Synthesis Technologies*, Durham, NC: Semiconductor Research Corporation, Arlington, VA, 2017, p. 39.

[btad572-B4] Bornholt J , Lopez R, Carmean DM et al A DNA-based archival storage system. In*: Proceedings of the Twenty-First International Conference on Architectural Support for Programming Languages and Operating Systems, ASPLOS ’16*. New York, NY: ACM, Atlanta, GA, 2016, 637–49. ISBN 978-1-4503-4091-5.

[btad572-B5] Chaykin G , Furman N, Sabary O et al DNA-Storalator: End-to-End DNA Storage Simulator. In: *Non-Volatile Memories Workshop 2022*, Oakland, CA: University of Calidfornia, San Diego, CA, 2022.

[btad572-B6] Choi Y , Ryu T, Lee AC et al High information capacity DNA-based data storage with augmented encoding characters using degenerate bases. Sci Rep 2019;9:6582–7. 31036920 10.1038/s41598-019-43105-w PMC6488701

[btad572-B7] Church GM , Gao Y, Kosuri S. Next-generation digital information storage in DNA. Science 2012;337:1628. 22903519 10.1126/science.1226355

[btad572-B8] Edgar RC. MUSCLE: a multiple sequence alignment method with reduced time and space complexity. BMC Bioinformatics 2004;5:113. 15318951 10.1186/1471-2105-5-113 PMC517706

[btad572-B9] Erlich Y , Zielinski D. DNA fountain enables a robust and efficient storage architecture. Science 2017;355:950–4. 28254941 10.1126/science.aaj2038

[btad572-B10] Goldman N , Bertone P, Chen S et al Towards practical, high-capacity, low-maintenance information storage in synthesized DNA. Nature 2013;494:77–80. 23354052 10.1038/nature11875 PMC3672958

[btad572-B11] Grass RN , Heckel R, Puddu M et al Robust chemical preservation of digital information on DNA in silica with error-correcting codes. Angew Chem Int Ed Engl 2015;54:2552–5. 25650567 10.1002/anie.201411378

[btad572-B12] Hamoum B , Dupraz E, Conde-Canencia L et al Channel model with memory for DNA data storage with nanopore sequencing. In: *2021 11th International Symposium on Topics in Coding (ISTC)*, New York, NY: IEEE, Quebec, Canada, 2021, 1–5.

[btad572-B13] Heckel R , Shomorony I, Ramchandran K et al Fundamental limits of DNA storage systems. In: *2017 IEEE International Symposium on Information Theory (ISIT)*, New York, NY: IEEE, Aachen, Germany, 2017, 3130–3134. ISSN: 2157–8117.

[btad572-B14] Lin KN , Volkel K, Tuck JM et al Dynamic and scalable DNA-based information storage. Nat Commun 2020;11:2981. 32532979 10.1038/s41467-020-16797-2 PMC7293219

[btad572-B15] Marelli A , Chiozzi T, Battistini N et al Integrating FPGA acceleration in the DNAssim framework for faster DNA-based data storage simulations. Electronics 2023;12:2621.

[btad572-B16] Matange K , Tuck JM, Keung AJ. DNA stability: a central design consideration for DNA data storage systems. Nat Commun 2021;12:1358. 33649304 10.1038/s41467-021-21587-5 PMC7921107

[btad572-B17] Organick L , Ang SD, Chen Y-J et al Random access in large-scale DNA data storage. Nat Biotechnol 2018;36:242–8. 29457795 10.1038/nbt.4079

[btad572-B18] Organick L , Chen Y-J, Dumas Ang S et al Probing the physical limits of reliable DNA data retrieval. Nat Commun 2020;11:616. 32001691 10.1038/s41467-020-14319-8 PMC6992699

[btad572-B19] Press WH , Hawkins JA, Jones SK et al HEDGES error-correcting code for DNA storage corrects indels and allows sequence constraints. Proc Natl Acad Sci U S A 2020;117:18489–96. 32675237 10.1073/pnas.2004821117 PMC7414044

[btad572-B20] Rashtchian C , Makarychev K, Racz M et al Clustering billions of reads for DNA data storage. In: Guyon I (ed.), Advances in Neural Information Processing Systems 30. Red Hook, NY: Curran Associates Inc, 2017, 3360–71.

[btad572-B21] Sabary O , Orlev Y, Shafir R et al SOLQC: synthetic oligo library quality control tool. Bioinformatics 2021;37:720–2. 32840559 10.1093/bioinformatics/btaa740

[btad572-B22] Tomek KJ , Volkel K, Simpson A et al Driving the scalability of DNA-based information storage systems. ACS Synth Biol 2019;8:1241–8. 31117362 10.1021/acssynbio.9b00100

[btad572-B23] Tomek KJ , Volkel K, Indermaur EW et al Promiscuous molecules for smarter file operations in DNA-based data storage. Nat Commun 2021;12:3518. 34112775 10.1038/s41467-021-23669-w PMC8192770

[btad572-B24] Yazdi SMHT , Gabrys R, Milenkovic O. Portable and error-free DNA-based data storage. Sci Rep 2017;7:5011. 28694453 10.1038/s41598-017-05188-1 PMC5503945

[btad572-B25] Yuan L , Xie Z, Wang Y et al DeSP: a systematic DNA storage error simulation pipeline. BMC Bioinformatics 2022;23:185. 35581548 10.1186/s12859-022-04723-w PMC9116035

