# Supplementary material for: FrameD: framework for DNA-based data storage design, verification, and validation
Source: Bioinformatics. 2023 Sep 15;39(10):btad572. doi: 10.1093/bioinformatics/btad572 (PMC10563143; doi:10.1093/bioinformatics/btad572)
Supplement: btad572_Supplementary_Data [file btad572_supplementary_data.pdf]

Supplementary Information

FrameD Index Support

As previously explained, FrameD will provide automatic indexing support for outer codes if simple incremental counting indexes are used. To illustrate this, we provide a schematic in Figure 1 of the indexing that FrameD provides. Initially, FrameD breaks down a file into a set of packets. These packets are broken down into sub-packets. The number of sub-packets is determined by the number of base-sequences in the original packet, and how many base-sequences should be in each sub-packet, both of which can be specified by the user. At each sub-packet level a user specifies an outer encoding pass. The outer encoding pass provides protection to the initial sub-packets consisting of data by generating new sub-packets called **ECC Sub-Packets**. The manner in which ECC Sub-Packets are constructed is determined by the user's outer code such as XOR or Reed-Solomon. The process of sub-packeting is supported to occur an arbitrary number of times, where are each sub-packeting level more error correction is introduced to protect the smaller sub-packets. Once ECC sub-packets are generated, they are treated as any other sub-packet from the same level with respect to the following sub-packeting steps. Eventually after splitting sub-packets from each level, there will be a point reached

Table 1. Table indicating the approach that current DNA storage systems can take in order to utilize FrameD. To condense explanation, we use notations to generally represent the steps of information conversion that prior works take. We allow $\mathcal{B}^x_y$ to represent a set of length y base x integers, e.g $B^3_2 \subseteq \{0,1\}^3$. To differentiate DNA information from digital domain information, we let $\mathcal{D}^x_y$ to represent length x DNA strings of base y. Allowing for bases $y \neq 4$ is necessary of composite DNA works (Choi et al., 2019; Anavy et al., 2019). We abbreviate Reed Solomon codes over field F with $RS(F)$, and represent base changes of information as $\Psi(\mathcal{B}^{x_0}_{y_0};\mathcal{B}^{x_1}_{y_1})$, where $\Psi(\cdot;\cdot)$ is a bijection map between the two sets of integers. To allow for variable length base changes used by Huffman codes, we can let x be a sequence of integers. For example, $\mathcal{B}^{\{3,4\}}_2 \subseteq \{0,1\}^3 \cup \{0,1\}^4$ is a set consisting of both length 3 and 4 base-2 integers. $\mathbf{P}$ and $\mathbf{I}$ indicate transformations applied to Payload/Index respectively.

	Single Strand			Multi Strand		
Storage System	Binary Transformation	Transcoding	Functional Site Encoding	Outer Code	Consolidation	Reconstruction
Bornholt et al. (2016)	Parity Check, $\Psi(\mathcal{B}^8_2;\mathcal{B}^{\{5,6\}}_3)$	$\Psi(\mathcal{B}^1_3;\mathcal{D}^1_4)$ Rotating Code	Prepend & Append Primers	Overlap Repetition, XOR	Detect & Remove	Index
Goldman et al. (2013)	Parity Check, $\Psi(\mathcal{B}^8_2;\mathcal{B}^{\{5,6\}}_3)$	$\Psi(\mathcal{B}^1_3;\mathcal{D}^1_4)$ Rotating Code	N/A	Overlap Repetition	Detect & Remove	Index
Tomek et al. (2019)	$\Psi(\mathcal{B}^8_2;\mathcal{B}^{\{5,6\}}_3)$	$\Psi(\mathcal{B}^1_3;\mathcal{D}^1_4)$ Rotating Code	Prepend & Append Primers	Overlap Repetition, XOR	Detect & Remove	Index
Lin et al. (2020)	N/A	Byte-to-DNA Map	Prepend & Append Primers, T7 Promoter	N/A	N/A	Index
Grass et al. (2015)	$\Psi(\mathcal{B}^2_{2^8};\mathcal{B}^3_{47})$, $RS(47)$	$\Psi(\mathcal{B}^1_{47};\mathcal{D}^3_4)$	Prepend & Append Primers	$\Psi(\mathcal{B}^2_{2^8};\mathcal{B}^3_{47})$, $RS(47^{39})$	N/A	Index
Press et al. (2020)	N/A	HEDGES Convolutional Code	Prepend & Append Primers	$RS(2^8)$	Detect & Remove	Index
Erlich and Zielinski (2017)	$RS(2^8)$	$\Psi(\mathcal{B}^1_2;\mathcal{D}^1_4)$	Prepend & Append Primers	Fountain Code	Detection & Remove	Luby Seed
Antkowiak et al. (2020)	Randomize, $RS(2^6)$	$\Psi(\mathcal{B}^1_2;\mathcal{D}^1_4)$	N/A	$RS(2^{14})$	DNA Cluster and MSA	Index
Choi et al. (2019)	N/A	$\mathbf{P}(\Psi(\mathcal{B}^7_2;\mathcal{D}^3_6)$, Nuc. Deduction), $\mathbf{I}(\Psi(\mathcal{B}^1_{48};\mathcal{D}^3_4))$	Prepend & Append Primers	$RS(2^7)$	N/A	Index
Anavy et al. (2019)	$\mathbf{P}(\Psi(\mathcal{B}^5_2;\mathcal{B}^2_6)$, $RS(7^3))$ $\mathbf{I}(RS(2^4))$	$\mathbf{P}(\Psi(\mathcal{B}^2_6;\mathcal{D}^2_6)$, Nuc. Deduction), $\mathbf{I}(\Psi(\mathcal{B}^2_2;\mathcal{D}^1_4))$	Prepend & Append Primers	Fountain	N/A	Luby Seed
Yazdi et al. (2017)	N/A	$\Psi(\mathcal{B}^{14}_2;\mathcal{D}^8_4)$ GC-balance Constrained Code with homopolymer checks	N/A	N/A	DNA Cluster and MSA	Index
Organick et al. (2018)	Randomize, $\Psi(\mathcal{B}^6_2;\mathcal{B}^4_3)$	$\Psi(\mathcal{B}^1_3;\mathcal{D}^1_4)$ Rotating Code	Prepend & Append Primers	$RS(2^{16})$	DNA Cluster and MSA	Index
Tomek et al. (2021)	$RS(2^8)$	$\Psi(\mathcal{B}^8_2;\mathcal{D}^8_4)$	Prepend & Append Primers, Restriction Enzymes	$RS(2^8)$	Detect & Remove	Index

where sub-packets are individual base-sequences. While ECC base-sequences can be added at this final level, no more sub-packeting is possible.

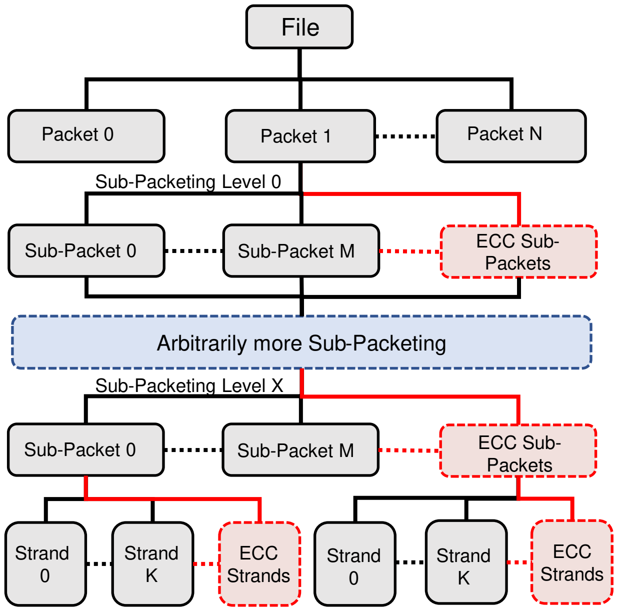

Fig. 1: Hierarchy that FrameD uses in order to allow arbitrary cascading of outer codes. During the outer code encoding process, FrameD infers index pieces to generate a complete unique identifier for each base-sequence.

FrameD handles the communication of information between sub-packet levels, really all the user needs to provide for an outer code is the number of ways a packet should be divided and algorithm to generate the ECC sub-packets given some starting data packets. If a user does not specify a terminating sub-packet level where the sub-packets are strands, FrameD will insert it automatically. While information is relayed between each level of the sub-packeting hierarchy, FrameD adds to each base-sequence the indexing information indicating what packet or sub-packet it is from. This indexing information is simply derived from a counter at each level that uniquely identifies each unit of information. Ultimately, the entire index consists of several smaller indexes, starting with the packet index and ending with the strand index. Consider a system that has 1 sub-packet level and a terminal strand sub-packet level. A base-sequence is uniquely identified by a 3-tuple of integers, with 1 integer for the packet, sub-packet, and strand. So, in general, for a base-sequence that is the kth strand of the jth sub-packet of the ith packet the index is (i, j, k). For outer code schemes that do not utilize a counter but rather use a random seed value to indicate data positions (fountain codes), they can still be supported by FrameD given that we allow any outer code to modify the index related to it's sub-packet level. However, such modification should be reversed on decoding to ensure that FrameD can reason about the resulting sub-packets and their ordering.

Decoding Details

For the most part, decoding in a DNA storage system is identical to encoding with the exception that the flow of information through passes is reversed. However, decoding has an additional problem that needs to be rectified, and that is how to reduce the dimensions of the input DNA data set that will typically consist of multiple reads per encoded DNA strand. There are two general approaches that can be taken. One is to first cluster the DNA strands input to the decoder based on similarity scores like edit distance or using a MinHash-based approaches (Antkowiak et al., 2020; Organick et al., 2018; Rashtchian et al., 2017). Then MSA algorithms, such as Muscle, can be used to aggregate information across strands and help resolve errors through consensus voting (Antkowiak et al., 2020; Edgar, 2004; Yazdi et al., 2017). Because clustering and alignment are usually discrete steps, we build a sub-model for DNA consolidation as shown in Figure 2.

Another approach is to consolidate the strands after completing the inner code, throwing out strands that may violate error checks, and coming to a consensus on the digital representation of information. FrameD supports the use of either approach, or even both. The only real difference between this approach and a DNA-based approach is that it would be placed after each strand is passed through inner decoding in Figure 2. Also, the clustering in this case is also trivial given that indexes are known after the inner decode passes.

Detailed Probe Example

Figure 3 provides a detailed illustration of an implementation of a probe that calculates error rate in terms of edit operations by way of calculating the edit distance between a fault injected DNA strand and its pre-injected version. In this illustration, we represent the state of a strand that is being manipulated as a **Strand Object**. This object holds attributes that represent pieces of a strand's information, such as its binary information and DNA information representations. The **DNA** field is initially empty until the strand passes through the Transcoding's encoding pass (top of Figure 3) which populates this field. After which, the edit distance probe takes a snapshot of this field and generates a copy that is placed in an attribute **DNA′** that can be referenced at a later time.

At the bottom of Figure 3 is the decoding pass which is applied after faults have been injected into DNA strands. The injected errors are represented by the red-highlighted letters in the **Strand Object**'s **DNA** attribute. Note that the **DNA′** is still in the **Strand Object**, and not modified. When this **Strand Object** passes through the **Edit Dist. Probe**, the probe is able to compare the now corrupted **DNA** field with its non-corrupted version in **DNA′** to determine the errors and their locations. This data can be transferred to FrameD's statistic tracking support which allows for the statistics to be propagated to the simulation's output files.

Decode Pipeline MPI Communication Pattern

In previous sections, we explained what units of work FrameD finds for parallelization using MPI. While FrameD handles the movement of information to facilitate parallelization for many steps of the decode process, clustering is a step in which we actually provide the user the flexibility to write their own communication pattern. For a user to write their own communication patters, it is necessary to understand the

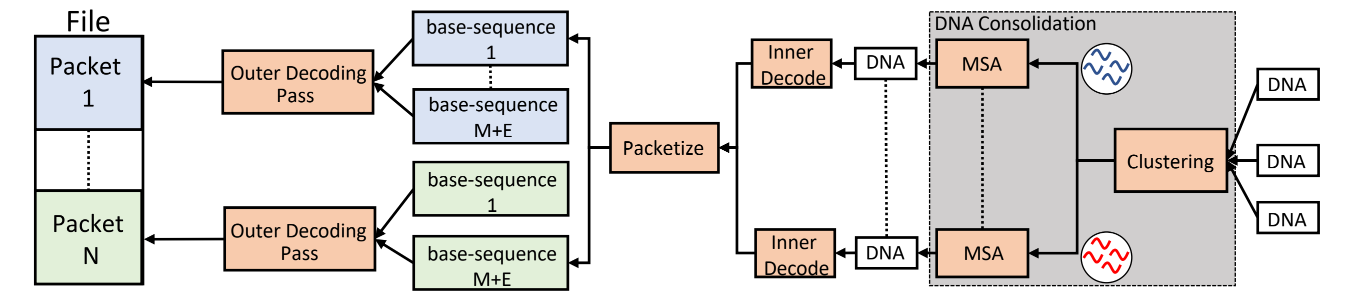

Fig. 2: Model of decoding for FrameD. Decoding is typically just the reversal of the encoding process, but DNA storage systems must address strand copy numbers. In this example, copies are consolidated by clustering the DNA strands and performing multi-sequence-alignment (MSA) on the result. In this figure Inner Decode represents the reverse operation of the Inner Encoding of Figure 1.

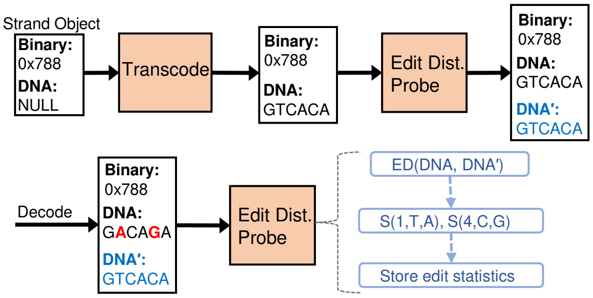

Fig. 3: Example of generating edit rate statistics in FrameD.

surrounding communication patterns to this step so that data is in an appropriate state for each rank. Figure 4 illustrates the transformation and communication of information from the time of instantiating a decode pipeline to the final point of writing packets to a file.

FrameD starts with all information in its original DNA state in Rank 0. This information is initially scattered across all ranks in the MPI communicator allocated for the decoding pipeline. The strands allocated to each rank are initially passed through a process that reverses the DNA modification steps. This is done first so that regions like primers that may indicate a certain file can be filtered, and so that other possible inserted DNA regions can be removed before reverse-transcoding. There are now two scenarios for processing the DNA strands, either clustering is done or it is not. If it is not, timeline of Figure 4 skips the steps in between the orange dashed lines which are only used when clustering DNA strands. In this case, the inner decode steps are processed in parallel for each DNA strand. After all strands are decoded for each rank, they are gathered into Rank 0 which places each base-sequence in its appropriate packet as indicated by the base-sequence's index. After packetizing the individual pieces of information of each DNA strand, the packets are scattered across ranks where each packet is decoded using the outer code. Finally, all packets are gathered back at Rank 0 so that information can be written to a file to output the information stored in DNA.

The steps in between the dashed orange line of Figure 4 correspond to the steps within the DNA-consolidation model of clustering and subsequent multi-sequence alignment. Initially, strands are gathered at Rank 0. We do this before clustering begins for several reasons. One, while we allow parallel clustering algorithms to be written for FrameD users may also want to write serial clustering algorithms while still utilizing FrameD's automatic parallelization. So, all strands will need to be on a single rank that will run the code for clustering, and in this case we assume Rank 0 for that role. Second, it establishes simple assumption that the user can make about the whereabouts of strands when they want to distribute strands according to their algorithm. Third, the implicit distribution of strands may not even be appropriate for a user's parallel clustering algorithm. Within the clustering algorithm, developers are free to utilize the provided MPI communicator in any way that they need to communicate information. After clustering, clusters should be placed in Rank 0 so that they are in a location that FrameD is aware of. Finally, given clusters represent a single DNA strand and a single piece of independent information, FrameD scatters the clusters before they are processed with MSA algorithms. The resulting strands at each rank from MSA are kept at each rank before the inner decode process since there is no need to gather and scatter again.

Read and Write Cost Methodology

We evaluate the write and read cost of all of the studied pipelines from an information density standpoint. That is, we assume that the read cost and write cost of the system is solely determined by the density in which data can be sequenced and synthesized. We make this assumption because at the moment, the cost to sequence and synthesize data is the main cost factor of a DNA storage system. We recognize that supporting infrastructure will be necessary for a DNA storage system, like compute to implement the decode process at a desired throughput. However, the wide range of possible algorithms, their unique complexities, and possibly different compute paradigms (single-thread/multi-thread/GPU), makes it difficult to provide a complete cost analysis that factors in compute. Furthermore, performing an information-based analysis may provide insight into possible directions in which future computational research should emphasize so that approaches with good information density can be achieved computationally efficiently.

While FrameD allows for parameter sweeps for encodings to optimize error rates, it is computationally inefficient to determine exactly the required outer code error correction for a given error rate and sequencing depth. So, we use results from targeted fault injection runs to build a simple analytical model that provides the probability of decoding a file for a given outer code and a given strand drop out rate if desired. From this, we pick outer

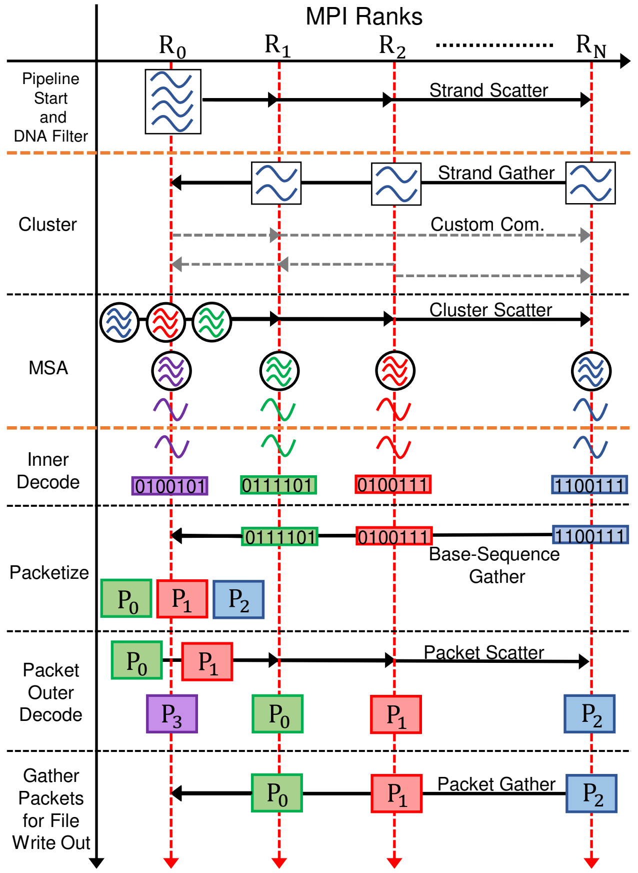

Fig. 4: Timing diagram of the representation of information in FrameD along with communication patterns used to parallelize the decode process using MPI. Events are ordered vertically in time for each MPI rank (dashed red lines) and are described on the left side between black/orange dashed lines. Items on each red line indicate the representation of information at a point in time. Curved lines indicate DNA representation, rectangular boxes with binary numbers indicate binary base-sequences, and rectangles labeled P_x indicate a packet of binary information.

code parameters that force this probability towards 1. Because approaches that use MSA or detect and throw out erroneous strands treat sequencing depth differently, we do two separate analyses.

We first begin with an approach that detects erroneous strands and removes them from decoding. Let $p(\text{drop-out})$ be the probability that an encoded strand does not get sequenced, and let $p(\text{decode}|e)$ be the probability that a strand decodes for a given error rate e. Assuming a constant sequencing depth d for a given strand that does not drop out, let n be the number of strands successfully decoded from the sequencing depth, then $p(n > 0|d, p(\text{decode}|e)) = 1 - (1 - p(\text{decode}|e))^d$ is the probability at least 1 strand is decoded successfully. Factoring in strand drop outs,the total probability that a strand with index I makes it to the outer decoder is $p(\text{I exists}|e, d, p(\text{drop-out})) = p(n > 0|d, p(\text{decode}|e)) \cdot (1 - p(\text{drop-out}))$. Assuming an outer code like Reed Solomon, and given D data symbols in a given encoded codeword, and all indexes that are not decoded successfully are correctly detected, then the outer code will succeed as long as at least D symbols are recovered from the total $N = D + E$ symbols where E is the additional error correction added to the set. The number of recovered symbols M will be distributed according to a Binomial distribution $B(N, p(\text{I exists}))$. Thus the probability that a block decodes is:

$$p(M \geq D) = 1 - \sum_{k=0}^{D-1} \binom{N}{k} p(\text{I exists})^k (1 - p(\text{I exists}))^{N-k} \quad (1)$$

So, for any file with X blocks, the probability to decode a file is succeeding on each block that is decoded independently, e.g. $p(\text{File decodes}) = p(M \geq D)^X$. From this analysis, we can see that there are two main unknowns. One is $p(\text{decode}|e)$ which depends both on the error rate and the decoding algorithm used. This value is the main target of our fault injection studies for each given pipeline. Another unknown is d, the number of reads per strand. In our analysis we assume that every strand that is not dropped out has exactly d reads. This is different than modeling some distribution over d, but we choose to assume a constant d for each strand because this provides a more accurate assessment of the amount of information that will need to be read during sequencing. Furthermore, distributions on the number of reads per strand is very process dependent (Bornholt et al., 2016; Organick et al., 2018; Tomek et al., 2019; Organick et al., 2020), and so an assumption of a distribution here may not provide generally useful results. To finally determine d, we sweep over a range of values to a get a set of $p(\text{I exists})$. With each of these probabilities we then sweep over a range of Reed-Solomon configurations each with a different value for E and D. We ultimately pick the smallest E such that the file can be recovered with a certain mean time to failure (MTTF). In our analyses we fix the file size to be $1MB$ and MTTF to be 10^9 reads. The MTTF for some number of blocks and Reed-Solomon configuration is defined as:

$$\text{MTTF} = \frac{1}{1 - p(M \geq D)^X} \quad (2)$$

For an approach using MSA, the outlined approach stays the same except that taking into account sequencing depth becomes different. Now, instead of individual reads being independently decoded, their information is aggregated using a MSA algorithm, so the probability that an index exists will be $p(\text{I exists}|e, d, p(\text{drop-out}), \text{MSA}) = p(\text{decode}|e, \text{MSA}, d)) \cdot (1 - p(\text{drop-out}))$. Where $p(\text{decode}|e, \text{MSA}, d)$ is the probability of successfully decoding d reads that have been aligned via MSA.

The outlined approaches provide a pathway to estimating the number of strands that are encoded and also the read depth for each encoded strand. However, to compare costs of different approaches, we normalize to a *bits/base* value for both reading and writing. This normalization is necessary because read costs are impacted not only by depth of sequencing, but also by the density of the encoding as well since this impacts total strands in the set of strands read. We define the write density with Equation 3 and the read density with Equation 4, where read density can be written as write density divided by the number of reads made per strand d:

$$\text{Write Density} = \frac{|F|}{\text{Total DNA Strands} \times \text{Length of DNA Strand}} \quad (3)$$

$$\text{Read Density} = \frac{\text{Write Density}}{d} \quad (4)$$

In Equation 3, $|F|$ represents the total number of bits that are encoded in some set of DNA strands.

Reed Solomon Inner Codes

To demonstrate why Reed Solomon inner code configurations that are less dense do not appear in Figure 4, we use Figure 5. In this figure we plot the write density that can be achieved for various strand decode probabilities. Each line represents the number of bytes of data that are allowed in an individual strand, and there is a line according to each configuration of **RS** in our experiment. Using these lines, we plot 4 points corresponding to write density and decode probabilities that have been observed for the 4 different density configurations when considering an i.i.d channel of 5% error rate. The blue star-point corresponds to configuring the Reed Solomon inner code with just error detection for a read depth of 10 reads per strand, and the other points correspond to all studied Reed Solomon inner code configurations for a read depth of 5 reads per strand.

As was pointed out in Figure 4, there is typically an eventual benefit in less dense inner encodings providing higher read densities when the read depth is decreased. A benefit in read density can come from 2 sources: increasing write density and decreasing read depth, as shown in Equation 4. Thus a less dense code can offset decreases in decode probability when read depth decreases, lowering the amount of outer code and subsequently increasing write density. Alternatively, a less dense code can also make lower read depth designs attainable with respect to MTTF metrics.

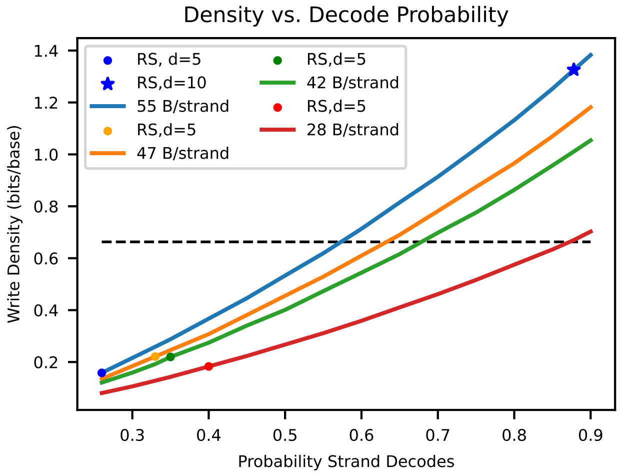

Fig. 5: Relationship between write density and the probability that an index exists for different amounts of bytes/strand.

However, this is not the case for the Reed Solomon inner code. The blue star-point of Figure 5 represents the rightmost point of Figure 4 for the 5% i.i.d channel. For this frontier to be extended, one of two things has to happen. Either a lower inner code density will provide better write density for the same read depth, in this case $d = 10$, or read depth is decreased. The former is not possible, given that the points in the frontier are optimal, a point not in the frontier that increases read and write density is not possible. Thus, we need to look towards smaller read depths. However, we found that as read depth decreases it is offset by extremely low write density for this pipeline. Given read density is the ratio of write density to read depth, an increase in read density is only possible if the ratio increase. This is illustrated by Figure 5, where we look at points that cut read depth in half from 10 to 5. Given a factor of 2 decrease in read depth, the write density must be higher than the black cut-off line that is placed at half the write density of $d = 10$. It is clear that every point for $d = 5$ falls under this cut-off, and thus no design is worthwhile. This showcases even more that Reed Solomon inner codes are not suitable for insertion/deletion channels and DNA data storage.

Additional Run Time Analysis

Here we provide more insight into the performance and runtime characteristics of all pipelines we simulated for this work. While in principle we could compare the parallel performance against that obtained on a single core, it would be intractable to run every pipeline on a single core sequentially. Instead, we analyze how uniformly each mpi-rank executes its assigned load measured by execution time. Each fault injection campaign logged key events such as when an iteration began and ended, and information such as the name of the host node where the rank executed.

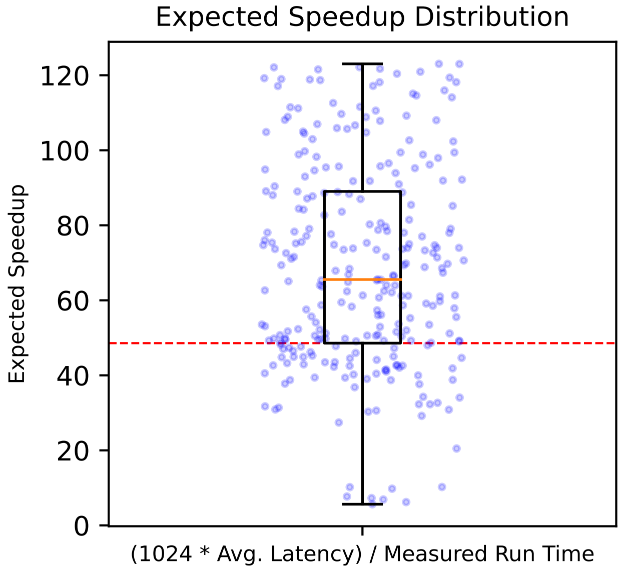

Fig. 6: Distribution of expected speedups calculated between average iteration latencies and the total measured run time. The red horizontal line plots the 25th percentile of the distribution.

Using the information within our logs, we calculated an average execution time across all 1024 iterations of a given pipeline. We also determine the difference between the start of the first iteration and the completion of the last iteration to finish and use this as the total time to complete all iterations. Using the average and the total difference in time for each pipeline, we calculate an *expected speedup* by multiplying the average by 1024 (total number of iterations) and dividing by the measured total difference in time. The distribution of this value is plotted in Figure 6. We found that

this statistic ranges in value from 5.63 to 123.04. Our scheduler allocated equal work to each rank at launch time (with 128 mpi-ranks and 8 iterations per rank). Speedups close to 128 indicate near-ideal distribution of work and performance scaling, while speedups much lower imply that some ranks performed far worse than average. Overall, we find that 75% have expected speedups over 48.57, and we conclude that many simulations benefited significantly from parallelization, but some inefficiencies remain and may benefit from additional optimization.

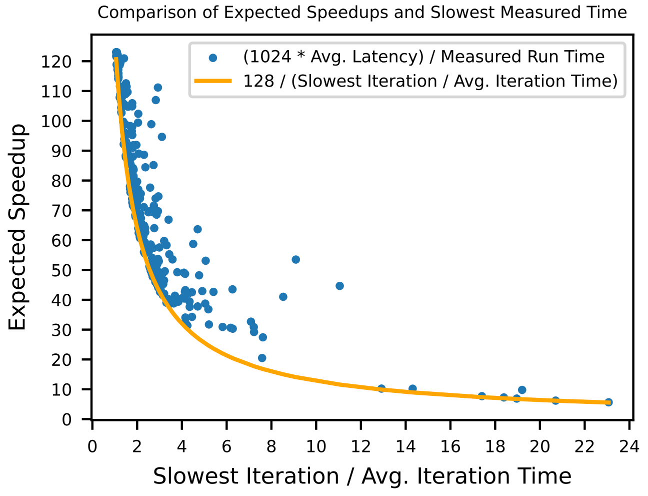

Fig. 7: Analysis of run times of all pipelines studied in this work.

The large variation in *expected speedup* could imply that some specific pipeline configurations perform poorly. However, we did not find any such correlation nor could we identify specific hosts in the cluster that consistently under-performed. Instead, we suspect the culprit is co-execution with other jobs on the cluster. We ran our simulations on shared compute resources used by many other research groups at NC State, and for our runs, we specified to the job scheduler that our mpi-ranks could be co-scheduled with another user's job. This can create scenarios where our job competes for CPU resources, such as cache, memory bandwidth, or memory capacity with other nodes. In such scenarios, one or both jobs scheduled on the node may be negatively impacted. To this end, we study the ranks that have the slowest iteration and determine its relationship with the expected speedups.

Figure 7 takes the data of Figure 6 and plots it against a value we calculate as Average Iteration/Slowest Iteration (blue points). From Figure 7 we can see that there is a strong inverse relationship between the expected speedup and this ratio. This provides evidence that slow ranks are a driving force in low expected speedups. To further investigate this, we take another approach to calculating an expected speedup. In this approach, we consider the ideal speedup of 128 and estimate how much we expect this to degrade by dividing by the ratio of Average Iteration/Slowest Iteration. The reasoning behind this approach is that it provides an estimate of what we expect the speedup to look like if at least one of the ranks executes all work at this slowest iteration pace. We plot this value in Figure 7 as a solid orange line. We see a significant clustering of blue points to the line, implying that slow ranks tend to stay slow.

Table 2. Analysis of the number of iterations per rank that fall in the 5% of iterations that have the longest execution time. The 5% slowest iterations are determined relative to each individual experiment.

Number of Iterations per Rank Executing Slowest 5% Iteration	Percentage of Total Ranks
0	86.71
1	5.84
2	1.94
3	1.15
4	0.99
5	0.68
6	0.49
7	0.48
8	1.73

Lastly, we consider whether these slow ranks are common. If they are outliers, it will imply that inefficiencies in our experiments are likely an artifact of the cluster we are using and that we may be able to improve scalability in these shared compute environments by updating the scheduler to avoid ranks that are executing slowly. To directly determine this, we take each of the ranks of a simulation and we determine the number of iterations within that rank that fall within the slowest 5% of all iterations. Table 2 summarizes this analysis for every rank of every simulation. Values in the first column indicate how many iterations that a rank executes which fall within the slowest 5%, and it varies from 0 to 8 since 8 iterations are assigned to each rank. The values in the second column report the percentage of all ranks that fall in that category. This data shows that a large majority of ranks (86.71%) do not execute any iterations that fall in the slowest 5%. However, we do find that there is a small group of ranks (1.73%) in which all 8 (the last row of the table) of their iterations are within the 5% slowest iterations. From this we conclude that inefficiencies in our experiment executions are likely caused by a small set of slow outlier ranks.

Thus, given the evidence that slow ranks tend to stay slow and the information that the slow ranks tend to be considerable outliers, we conclude that FrameD could see improvement by adapting the scheduler to allocate less work to ranks running slowly.
